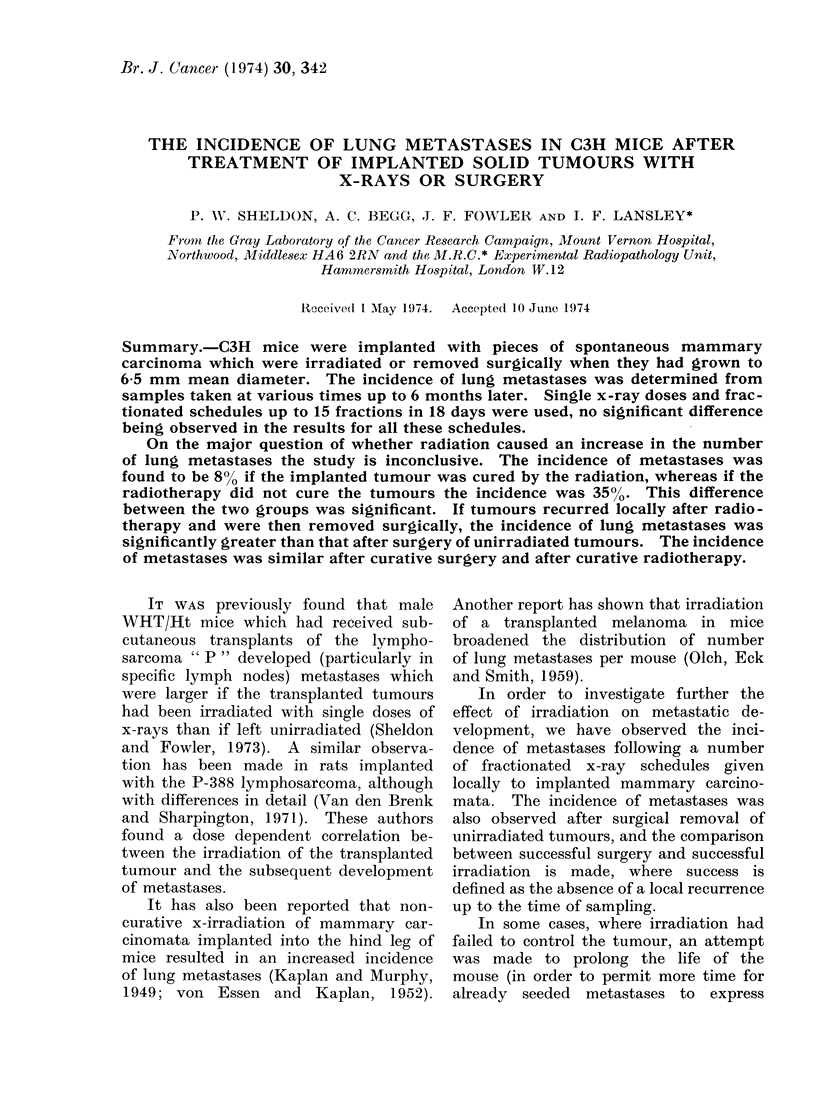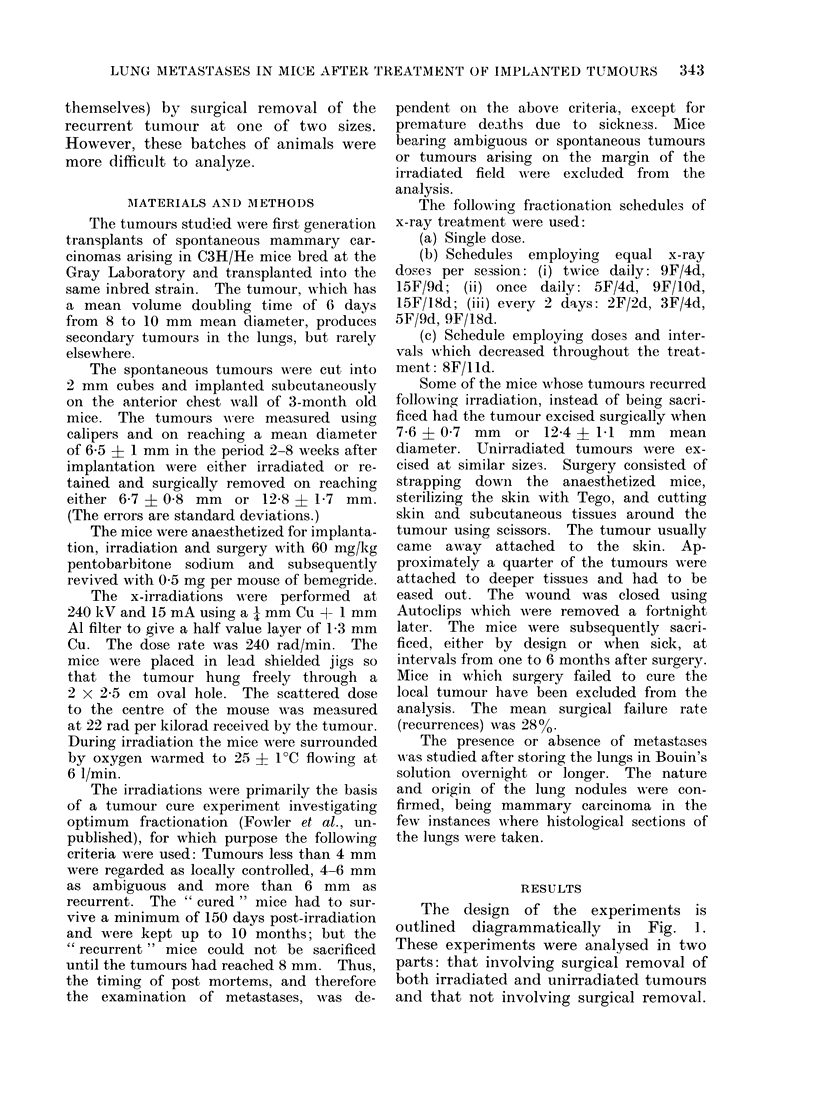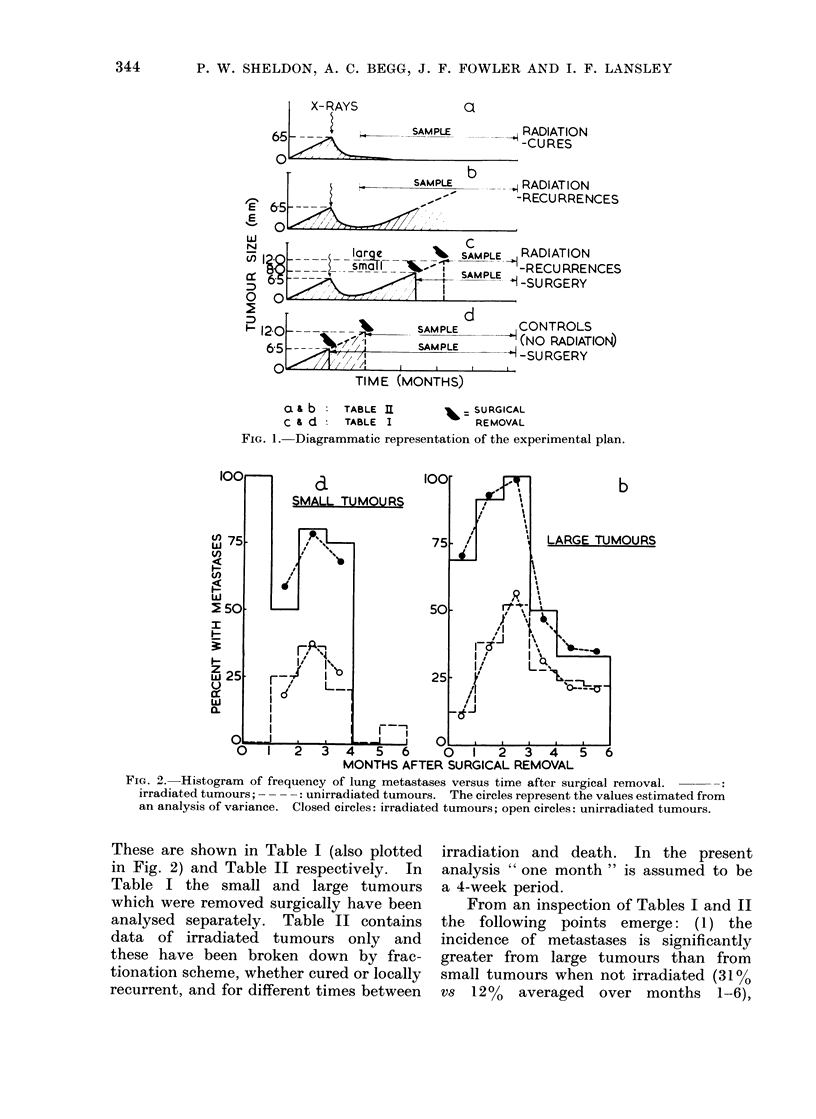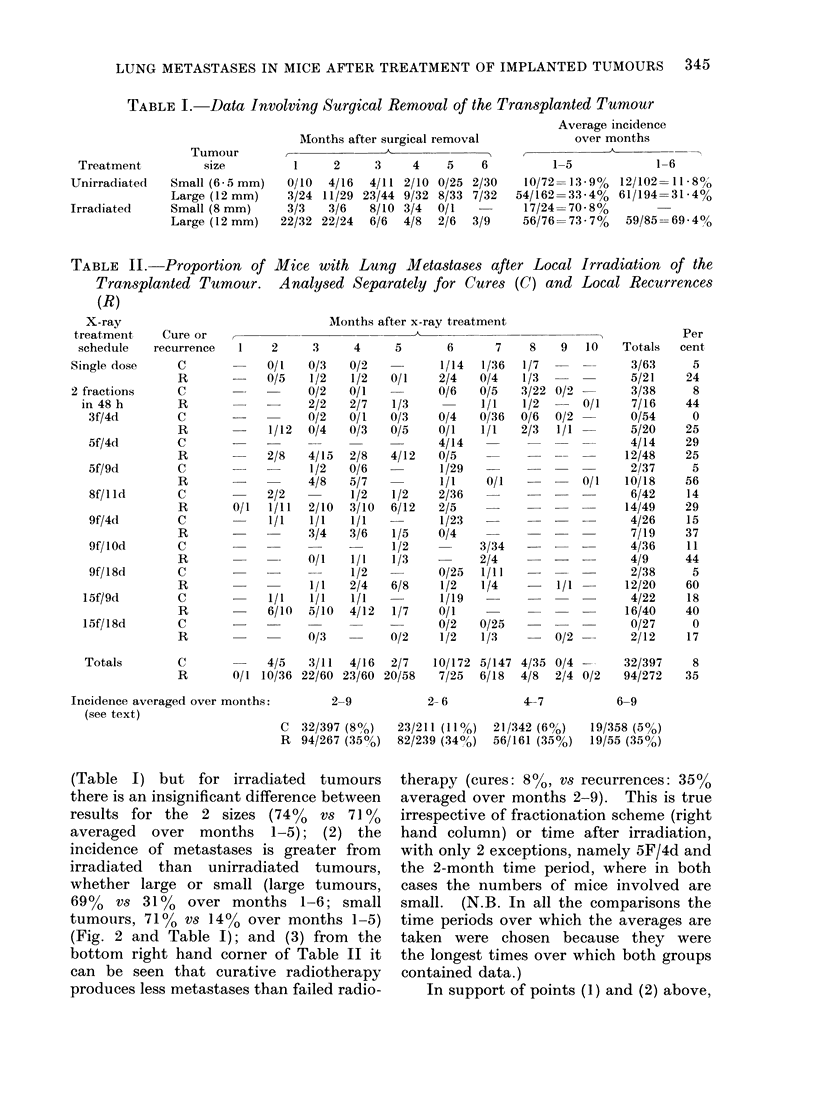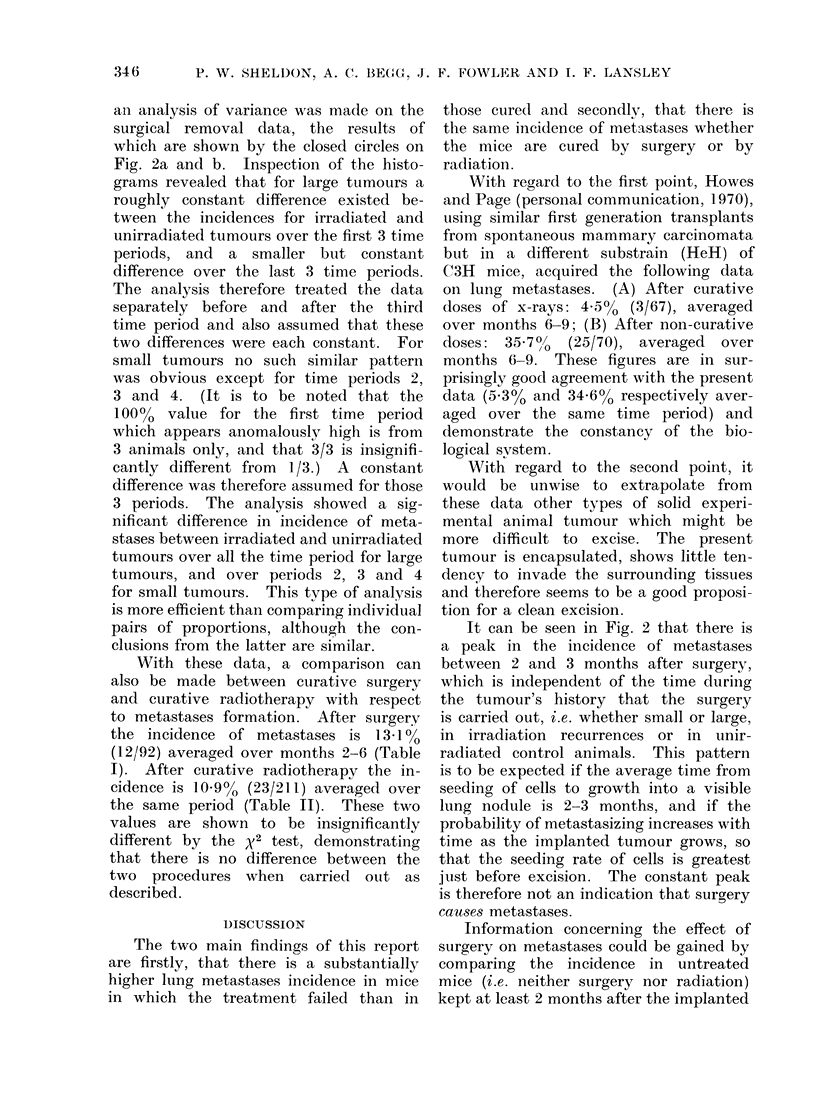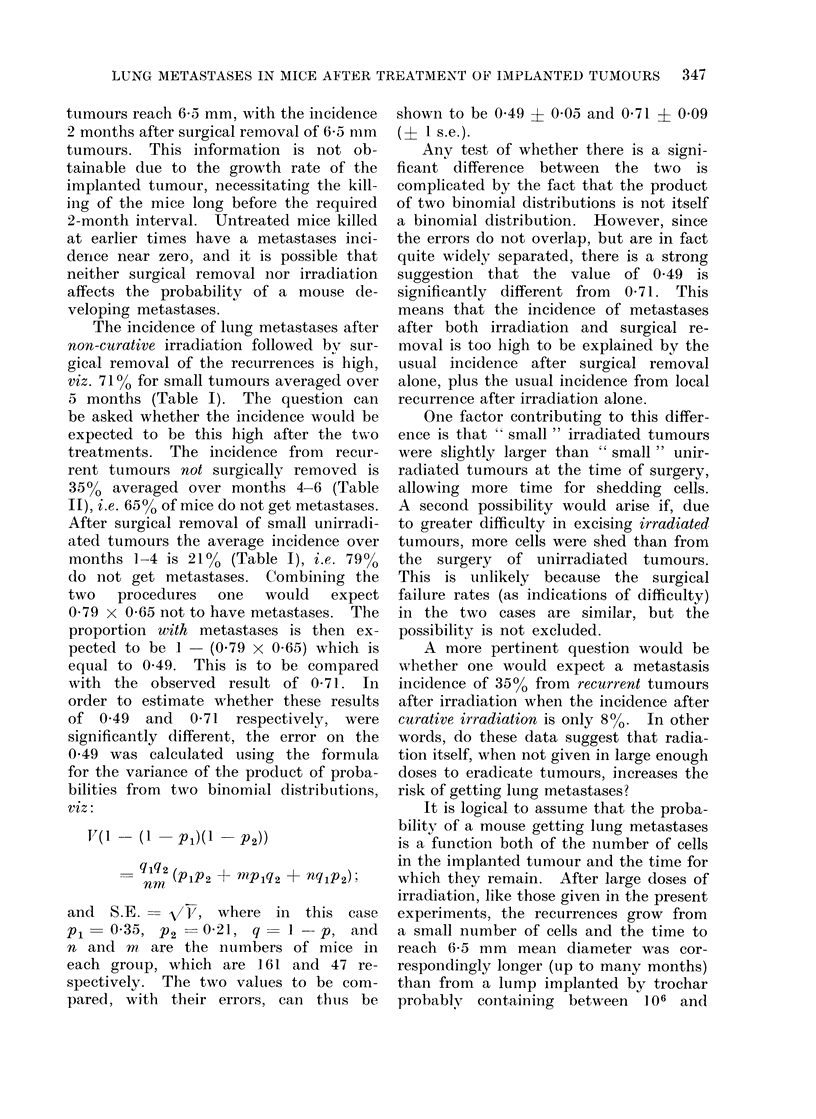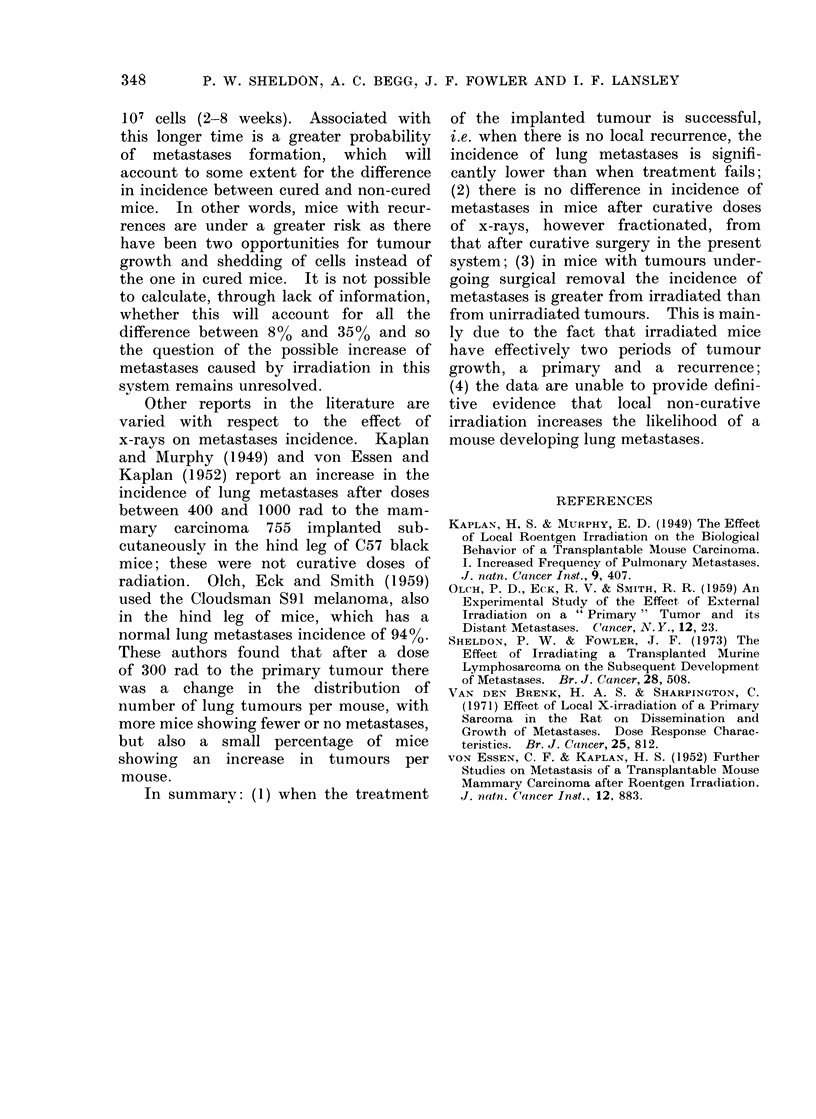# The Incidence of Lung Metastases in C3h Mice after Treatment of Implanted Solid Tumours with X-rays or Surgery

**DOI:** 10.1038/bjc.1974.203

**Published:** 1974-10

**Authors:** P. W. Sheldon, A. C. Begg, J. F. Fowler, I. F. Lansley

## Abstract

C3H mice were implanted with pieces of spontaneous mammary carcinoma which were irradiated or removed surgically when they had grown to 6·5 mm mean diameter. The incidence of lung metastases was determined from samples taken at various times up to 6 months later. Single x-ray doses and fractionated schedules up to 15 fractions in 18 days were used, no significant difference being observed in the results for all these schedules.

On the major question of whether radiation caused an increase in the number of lung metastases the study is inconclusive. The incidence of metastases was found to be 8% if the implanted tumour was cured by the radiation, whereas if the radiotherapy did not cure the tumours the incidence was 35%. This difference between the two groups was significant. If tumours recurred locally after radiotherapy and were then removed surgically, the incidence of lung metastases was significantly greater than that after surgery of unirradiated tumours. The incidence of metastases was similar after curative surgery and after curative radiotherapy.


*Br. J. Cancer* (1974) **30**, 342

### THE INCIDENCE OF LUNG METASTASES IN C3H MICE AFTER TREATMENT OF IMPLANTED SOLID TUMOURS WITH X-RAYS OR SURGERY

P. W. SHELDON, A. C. BEGG, J. F. FOWLER AND I. F. LANSLEY*

*From the Gray Laboratory of the Cancer Research Campaign, Mount Vernon Hospital, Northwood, Middlesex HA6 2RN and the M.R.C.* Experimental Radiopathology Unit, Hammersmith Hospital, London W.12*

Received 1 May 1974. Accepted 10 June 1974

**Summary.—C3H mice were implanted with pieces of spontaneous mammary carcinoma which were irradiated or removed surgically when they had grown to 6·5 mm mean diameter. The incidence of lung metastases was determined from samples taken at various times up to 6 months later. Single x-ray doses and fractionated schedules up to 15 fractions in 18 days were used, no significant difference being observed in the results for all these schedules.**

**On the major question of whether radiation caused an increase in the number of lung metastases the study is inconclusive. The incidence of metastases was found to be 8% if the implanted tumour was cured by the radiation, whereas if the radiotherapy did not cure the tumours the incidence was 35%. This difference between the two groups was significant. If tumours recurred locally after radiotherapy and were then removed surgically, the incidence of lung metastases was significantly greater than that after surgery of unirradiated tumours. The incidence of metastases was similar after curative surgery and after curative radiotherapy.**

IT WAS previously found that male WHT/Ht mice which had received subcutaneous transplants of the lymphosarcoma " P " developed (particularly in specific lymph nodes) metastases which were larger if the transplanted tumours had been irradiated with single doses of x-rays than if left unirradiated (Sheldon and Fowler, 1973). A similar observation has been made in rats implanted with the P-388 lymphosarcoma, although with differences in detail (Van den Brenk and Sharpington, 1971). These authors found a dose dependent correlation between the irradiation of the transplanted tumour and the subsequent development of metastases.

It has also been reported that noncurative x-irradiation of mammary carcinomata implanted into the hind leg of mice resulted in an increased incidence of lung metastases (Kaplan and Murphy, 1949; von Essen and Kaplan, 1952). Another report has shown that irradiation of a transplanted melanoma in mice broadened the distribution of number of lung metastases per mouse (Olch, Eck and Smith, 1959).

In order to investigate further the effect of irradiation on metastatic development, we have observed the incidence of metastases following a number of fractionated x-ray schedules given locally to implanted mammary carcinomata. The incidence of metastases was also observed after surgical removal of unirradiated tumours, and the comparison between successful surgery and successful irradiation is made, where success is defined as the absence of a local recurrence up to the time of sampling.

In some cases, where irradiation had failed to control the tumour, an attempt was made to prolong the life of the mouse (in order to permit more time for already seeded metastases to express themselves) by surgical removal of the recurrent tumour at one of two sizes. However, these batches of animals were more difficult to analyze.

### MATERIALS AND METHODS

The tumours studied were first generation transplants of spontaneous mammary carcinomas arising in C3H/He mice bred at the Gray Laboratory and transplanted into the same inbred strain. The tumour, which has a mean volume doubling time of 6 days from 8 to 10 mm mean diameter, produces secondary tumours in the lungs, but rarely elsewhere.

The spontaneous tumours were cut into 2 mm cubes and implanted subcutaneously on the anterior chest wall of 3-month old mice. The tumours were measured using calipers and on reaching a mean diameter of $6{\cdot}5 \pm 1$ mm in the period 2–8 weeks after implantation were either irradiated or retained and surgically removed on reaching either $6{\cdot}7 \pm 0{\cdot}8$ mm or $12{\cdot}8 \pm 1{\cdot}7$ mm. (The errors are standard deviations.)

The mice were anaesthetized for implantation, irradiation and surgery with 60 mg/kg pentobarbitone sodium and subsequently revived with 0·5 mg per mouse of bemegride.

The x-irradiations were performed at 240 kV and 15 mA using a $\frac{1}{4}$ mm Cu + 1 mm Al filter to give a half value layer of 1·3 mm Cu. The dose rate was 240 rad/min. The mice were placed in lead shielded jigs so that the tumour hung freely through a $2 \times 2{\cdot}5$ cm oval hole. The scattered dose to the centre of the mouse was measured at 22 rad per kilorad received by the tumour. During irradiation the mice were surrounded by oxygen warmed to $25 \pm 1$°C flowing at 6 l/min.

The irradiations were primarily the basis of a tumour cure experiment investigating optimum fractionation (Fowler *et al.*, unpublished), for which purpose the following criteria were used: Tumours less than 4 mm were regarded as locally controlled, 4–6 mm as ambiguous and more than 6 mm as recurrent. The "cured" mice had to survive a minimum of 150 days post-irradiation and were kept up to 10 months; but the "recurrent" mice could not be sacrificed until the tumours had reached 8 mm. Thus, the timing of post mortems, and therefore the examination of metastases, was dependent on the above criteria, except for premature deaths due to sickness. Mice bearing ambiguous or spontaneous tumours or tumours arising on the margin of the irradiated field were excluded from the analysis.

The following fractionation schedules of x-ray treatment were used:

(a) Single dose.

(b) Schedules employing equal x-ray doses per session: (i) twice daily: 9F/4d, 15F/9d; (ii) once daily: 5F/4d, 9F/10d, 15F/18d; (iii) every 2 days: 2F/2d, 3F/4d, 5F/9d, 9F/18d.

(c) Schedule employing doses and intervals which decreased throughout the treatment: 8F/11d.

Some of the mice whose tumours recurred following irradiation, instead of being sacrificed had the tumour excised surgically when $7{\cdot}6 \pm 0{\cdot}7$ mm or $12{\cdot}4 \pm 1{\cdot}1$ mm mean diameter. Unirradiated tumours were excised at similar sizes. Surgery consisted of strapping down the anaesthetized mice, sterilizing the skin with Tego, and cutting skin and subcutaneous tissues around the tumour using scissors. The tumour usually came away attached to the skin. Approximately a quarter of the tumours were attached to deeper tissues and had to be eased out. The wound was closed using Autoclips which were removed a fortnight later. The mice were subsequently sacrificed, either by design or when sick, at intervals from one to 6 months after surgery. Mice in which surgery failed to cure the local tumour have been excluded from the analysis. The mean surgical failure rate (recurrences) was 28%.

The presence or absence of metastases was studied after storing the lungs in Bouin's solution overnight or longer. The nature and origin of the lung nodules were confirmed, being mammary carcinoma in the few instances where histological sections of the lungs were taken.

### RESULTS

The design of the experiments is outlined diagrammatically in Fig. 1. These experiments were analysed in two parts: that involving surgical removal of both irradiated and unirradiated tumours and that not involving surgical removal.

FIG. 1.—Diagrammatic representation of the experimental plan.

FIG. 2.—Histogram of frequency of lung metastases versus time after surgical removal. ———: irradiated tumours; – – – –: unirradiated tumours. The circles represent the values estimated from an analysis of variance. Closed circles: irradiated tumours; open circles: unirradiated tumours.

These are shown in Table I (also plotted in Fig. 2) and Table II respectively. In Table I the small and large tumours which were removed surgically have been analysed separately. Table II contains data of irradiated tumours only and these have been broken down by fractionation scheme, whether cured or locally recurrent, and for different times between irradiation and death. In the present analysis " one month " is assumed to be a 4-week period.

From an inspection of Tables I and II the following points emerge: (1) the incidence of metastases is significantly greater from large tumours than from small tumours when not irradiated (31% *vs* 12% averaged over months 1–6),

TABLE I.—*Data Involving Surgical Removal of the Transplanted Tumour*

| Treatment | Tumour size | Months after surgical removal | | | | | | Average incidence over months | |
|---|---|---|---|---|---|---|---|---|---|
| | | 1 | 2 | 3 | 4 | 5 | 6 | 1–5 | 1–6 |
| Unirradiated | Small (6·5 mm) | 0/10 | 4/16 | 4/11 | 2/10 | 0/25 | 2/30 | 10/72 = 13·9% | 12/102 = 11·8% |
| | Large (12 mm) | 3/24 | 11/29 | 23/44 | 9/32 | 8/33 | 7/32 | 54/162 = 33·4% | 61/194 = 31·4% |
| Irradiated | Small (8 mm) | 3/3 | 3/6 | 8/10 | 3/4 | 0/1 | — | 17/24 = 70·8% | — |
| | Large (12 mm) | 22/32 | 22/24 | 6/6 | 4/8 | 2/6 | 3/9 | 56/76 = 73·7% | 59/85 = 69·4% |

TABLE II.—*Proportion of Mice with Lung Metastases after Local Irradiation of the Transplanted Tumour. Analysed Separately for Cures (C) and Local Recurrences (R)*

| X-ray treatment schedule | Cure or recurrence | Months after x-ray treatment | | | | | | | | | | Totals | Per cent |
|---|---|---|---|---|---|---|---|---|---|---|---|---|---|
| | | 1 | 2 | 3 | 4 | 5 | 6 | 7 | 8 | 9 | 10 | | |
| Single dose | C | — | 0/1 | 0/3 | 0/2 | — | 1/14 | 1/36 | 1/7 | — | — | 3/63 | 5 |
| | R | — | 0/5 | 1/2 | 1/2 | 0/1 | 2/4 | 0/4 | 1/3 | — | — | 5/21 | 24 |
| 2 fractions | C | — | — | 0/2 | 0/1 | — | 0/6 | 0/5 | 3/22 | 0/2 | — | 3/38 | 8 |
| in 48 h | R | — | — | 2/2 | 2/7 | 1/3 | — | 1/1 | 1/2 | — | 0/1 | 7/16 | 44 |
| 3f/4d | C | — | — | 0/2 | 0/1 | 0/3 | 0/4 | 0/36 | 0/6 | 0/2 | — | 0/54 | 0 |
| | R | — | 1/12 | 0/4 | 0/3 | 0/5 | 0/1 | 1/1 | 2/3 | 1/1 | — | 5/20 | 25 |
| 5f/4d | C | — | — | — | — | — | 4/14 | — | — | — | — | 4/14 | 29 |
| | R | — | 2/8 | 4/15 | 2/8 | 4/12 | 0/5 | — | — | — | — | 12/48 | 25 |
| 5f/9d | C | — | — | 1/2 | 0/6 | — | 1/29 | — | — | — | — | 2/37 | 5 |
| | R | — | — | 4/8 | 5/7 | — | 1/1 | 0/1 | — | — | 0/1 | 10/18 | 56 |
| 8f/11d | C | — | 2/2 | — | 1/2 | 1/2 | 2/36 | — | — | — | — | 6/42 | 14 |
| | R | 0/1 | 1/11 | 2/10 | 3/10 | 6/12 | 2/5 | — | — | — | — | 14/49 | 29 |
| 9f/4d | C | — | 1/1 | 1/1 | 1/1 | — | 1/23 | — | — | — | — | 4/26 | 15 |
| | R | — | — | 3/4 | 3/6 | 1/5 | 0/4 | — | — | — | — | 7/19 | 37 |
| 9f/10d | C | — | — | — | — | 1/2 | — | 3/34 | — | — | — | 4/36 | 11 |
| | R | — | — | 0/1 | 1/1 | 1/3 | — | 2/4 | — | — | — | 4/9 | 44 |
| 9f/18d | C | — | — | — | 1/2 | — | 0/25 | 1/11 | — | — | — | 2/38 | 5 |
| | R | — | — | 1/1 | 2/4 | 6/8 | 1/2 | 1/4 | — | 1/1 | — | 12/20 | 60 |
| 15f/9d | C | — | 1/1 | 1/1 | 1/1 | — | 1/19 | — | — | — | — | 4/22 | 18 |
| | R | — | 6/10 | 5/10 | 4/12 | 1/7 | 0/1 | — | — | — | — | 16/40 | 40 |
| 15f/18d | C | — | — | — | — | — | 0/2 | 0/25 | — | — | — | 0/27 | 0 |
| | R | — | — | 0/3 | — | 0/2 | 1/2 | 1/3 | — | 0/2 | — | 2/12 | 17 |
| Totals | C | — | 4/5 | 3/11 | 4/16 | 2/7 | 10/172 | 5/147 | 4/35 | 0/4 | — | 32/397 | 8 |
| | R | 0/1 | 10/36 | 22/60 | 23/60 | 20/58 | 7/25 | 6/18 | 4/8 | 2/4 | 0/2 | 94/272 | 35 |

| Incidence averaged over months: (see text) | 2–9 | 2–6 | 4–7 | 6–9 |
|---|---|---|---|---|
| C | 32/397 (8%) | 23/211 (11%) | 21/342 (6%) | 19/358 (5%) |
| R | 94/267 (35%) | 82/239 (34%) | 56/161 (35%) | 19/55 (35%) |

(Table I) but for irradiated tumours there is an insignificant difference between results for the 2 sizes (74% *vs* 71% averaged over months 1–5); (2) the incidence of metastases is greater from irradiated than unirradiated tumours, whether large or small (large tumours, 69% *vs* 31% over months 1–6; small tumours, 71% *vs* 14% over months 1–5) (Fig. 2 and Table I); and (3) from the bottom right hand corner of Table II it can be seen that curative radiotherapy produces less metastases than failed radiotherapy (cures: 8%, *vs* recurrences: 35% averaged over months 2–9). This is true irrespective of fractionation scheme (right hand column) or time after irradiation, with only 2 exceptions, namely 5F/4d and the 2-month time period, where in both cases the numbers of mice involved are small. (N.B. In all the comparisons the time periods over which the averages are taken were chosen because they were the longest times over which both groups contained data.)

In support of points (1) and (2) above, an analysis of variance was made on the surgical removal data, the results of which are shown by the closed circles on Fig. 2a and b. Inspection of the histograms revealed that for large tumours a roughly constant difference existed between the incidences for irradiated and unirradiated tumours over the first 3 time periods, and a smaller but constant difference over the last 3 time periods. The analysis therefore treated the data separately before and after the third time period and also assumed that these two differences were each constant. For small tumours no such similar pattern was obvious except for time periods 2, 3 and 4. (It is to be noted that the 100% value for the first time period which appears anomalously high is from 3 animals only, and that 3/3 is insignificantly different from 1/3.) A constant difference was therefore assumed for those 3 periods. The analysis showed a significant difference in incidence of metastases between irradiated and unirradiated tumours over all the time period for large tumours, and over periods 2, 3 and 4 for small tumours. This type of analysis is more efficient than comparing individual pairs of proportions, although the conclusions from the latter are similar.

With these data, a comparison can also be made between curative surgery and curative radiotherapy with respect to metastases formation. After surgery the incidence of metastases is 13·1% (12/92) averaged over months 2–6 (Table I). After curative radiotherapy the incidence is 10·9% (23/211) averaged over the same period (Table II). These two values are shown to be insignificantly different by the $\chi^2$ test, demonstrating that there is no difference between the two procedures when carried out as described.

### DISCUSSION

The two main findings of this report are firstly, that there is a substantially higher lung metastases incidence in mice in which the treatment failed than in those cured and secondly, that there is the same incidence of metastases whether the mice are cured by surgery or by radiation.

With regard to the first point, Howes and Page (personal communication, 1970), using similar first generation transplants from spontaneous mammary carcinomata but in a different substrain (HeH) of C3H mice, acquired the following data on lung metastases. (A) After curative doses of x-rays: 4·5% (3/67), averaged over months 6–9; (B) After non-curative doses: 35·7% (25/70), averaged over months 6–9. These figures are in surprisingly good agreement with the present data (5·3% and 34·6% respectively averaged over the same time period) and demonstrate the constancy of the biological system.

With regard to the second point, it would be unwise to extrapolate from these data other types of solid experimental animal tumour which might be more difficult to excise. The present tumour is encapsulated, shows little tendency to invade the surrounding tissues and therefore seems to be a good proposition for a clean excision.

It can be seen in Fig. 2 that there is a peak in the incidence of metastases between 2 and 3 months after surgery, which is independent of the time during the tumour's history that the surgery is carried out, *i.e.* whether small or large, in irradiation recurrences or in unirradiated control animals. This pattern is to be expected if the average time from seeding of cells to growth into a visible lung nodule is 2–3 months, and if the probability of metastasizing increases with time as the implanted tumour grows, so that the seeding rate of cells is greatest just before excision. The constant peak is therefore not an indication that surgery *causes* metastases.

Information concerning the effect of surgery on metastases could be gained by comparing the incidence in untreated mice (*i.e.* neither surgery nor radiation) kept at least 2 months after the implanted tumours reach 6·5 mm, with the incidence 2 months after surgical removal of 6·5 mm tumours. This information is not obtainable due to the growth rate of the implanted tumour, necessitating the killing of the mice long before the required 2-month interval. Untreated mice killed at earlier times have a metastases incidence near zero, and it is possible that neither surgical removal nor irradiation affects the probability of a mouse developing metastases.

The incidence of lung metastases after *non-curative* irradiation followed by surgical removal of the recurrences is high, *viz.* 71% for small tumours averaged over 5 months (Table I). The question can be asked whether the incidence would be expected to be this high after the two treatments. The incidence from recurrent tumours *not* surgically removed is 35% averaged over months 4–6 (Table II), *i.e.* 65% of mice do not get metastases. After surgical removal of small unirradiated tumours the average incidence over months 1–4 is 21% (Table I), *i.e.* 79% do not get metastases. Combining the two procedures one would expect 0·79 × 0·65 not to have metastases. The proportion *with* metastases is then expected to be 1 — (0·79 × 0·65) which is equal to 0·49. This is to be compared with the observed result of 0·71. In order to estimate whether these results of 0·49 and 0·71 respectively, were significantly different, the error on the 0·49 was calculated using the formula for the variance of the product of probabilities from two binomial distributions, *viz*:

$$V(1-(1-p_1)(1-p_2)) = \frac{q_1q_2}{nm}(p_1p_2 + mp_1q_2 + nq_1p_2);$$

and S.E. $= \sqrt{V}$, where in this case $p_1 = 0{\cdot}35$, $p_2 = 0{\cdot}21$, $q = 1 - p$, and $n$ and $m$ are the numbers of mice in each group, which are 161 and 47 respectively. The two values to be compared, with their errors, can thus be shown to be 0·49 ± 0·05 and 0·71 ± 0·09 (± 1 s.e.).

Any test of whether there is a significant difference between the two is complicated by the fact that the product of two binomial distributions is not itself a binomial distribution. However, since the errors do not overlap, but are in fact quite widely separated, there is a strong suggestion that the value of 0·49 is significantly different from 0·71. This means that the incidence of metastases after both irradiation and surgical removal is too high to be explained by the usual incidence after surgical removal alone, plus the usual incidence from local recurrence after irradiation alone.

One factor contributing to this difference is that " small " irradiated tumours were slightly larger than " small " unirradiated tumours at the time of surgery, allowing more time for shedding cells. A second possibility would arise if, due to greater difficulty in excising *irradiated* tumours, more cells were shed than from the surgery of unirradiated tumours. This is unlikely because the surgical failure rates (as indications of difficulty) in the two cases are similar, but the possibility is not excluded.

A more pertinent question would be whether one would expect a metastasis incidence of 35% from *recurrent* tumours after irradiation when the incidence after *curative irradiation* is only 8%. In other words, do these data suggest that radiation itself, when not given in large enough doses to eradicate tumours, increases the risk of getting lung metastases?

It is logical to assume that the probability of a mouse getting lung metastases is a function both of the number of cells in the implanted tumour and the time for which they remain. After large doses of irradiation, like those given in the present experiments, the recurrences grow from a small number of cells and the time to reach 6·5 mm mean diameter was correspondingly longer (up to many months) than from a lump implanted by trochar probably containing between $10^6$ and

$10^7$ cells (2–8 weeks). Associated with this longer time is a greater probability of metastases formation, which will account to some extent for the difference in incidence between cured and non-cured mice. In other words, mice with recurrences are under a greater risk as there have been two opportunities for tumour growth and shedding of cells instead of the one in cured mice. It is not possible to calculate, through lack of information, whether this will account for all the difference between 8% and 35% and so the question of the possible increase of metastases caused by irradiation in this system remains unresolved.

Other reports in the literature are varied with respect to the effect of x-rays on metastases incidence. Kaplan and Murphy (1949) and von Essen and Kaplan (1952) report an increase in the incidence of lung metastases after doses between 400 and 1000 rad to the mammary carcinoma 755 implanted subcutaneously in the hind leg of C57 black mice; these were not curative doses of radiation. Olch, Eck and Smith (1959) used the Cloudsman S91 melanoma, also in the hind leg of mice, which has a normal lung metastases incidence of 94%. These authors found that after a dose of 300 rad to the primary tumour there was a change in the distribution of number of lung tumours per mouse, with more mice showing fewer or no metastases, but also a small percentage of mice showing an increase in tumours per mouse.

In summary: (1) when the treatment of the implanted tumour is successful, *i.e.* when there is no local recurrence, the incidence of lung metastases is significantly lower than when treatment fails; (2) there is no difference in incidence of metastases in mice after curative doses of x-rays, however fractionated, from that after curative surgery in the present system; (3) in mice with tumours undergoing surgical removal the incidence of metastases is greater from irradiated than from unirradiated tumours. This is mainly due to the fact that irradiated mice have effectively two periods of tumour growth, a primary and a recurrence; (4) the data are unable to provide definitive evidence that local non-curative irradiation increases the likelihood of a mouse developing lung metastases.

### REFERENCES

KAPLAN, H. S. & MURPHY, E. D. (1949) The Effect of Local Roentgen Irradiation on the Biological Behavior of a Transplantable Mouse Carcinoma. I. Increased Frequency of Pulmonary Metastases. *J. natn. Cancer Inst.*, **9**, 407.

OLCH, P. D., ECK, R. V. & SMITH, R. R. (1959) An Experimental Study of the Effect of External Irradiation on a "Primary" Tumor and its Distant Metastases. *Cancer, N.Y.*, **12**, 23.

SHELDON, P. W. & FOWLER, J. F. (1973) The Effect of Irradiating a Transplanted Murine Lymphosarcoma on the Subsequent Development of Metastases. *Br. J. Cancer*, **28**, 508.

VAN DEN BRENK, H. A. S. & SHARPINGTON, C. (1971) Effect of Local X-irradiation of a Primary Sarcoma in the Rat on Dissemination and Growth of Metastases. Dose Response Characteristics. *Br. J. Cancer*, **25**, 812.

VON ESSEN, C. F. & KAPLAN, H. S. (1952) Further Studies on Metastasis of a Transplantable Mouse Mammary Carcinoma after Roentgen Irradiation. *J. natn. Cancer Inst.*, **12**, 883.